# Highly sensitive liquid biopsy Duplex sequencing complements tissue biopsy to enhance detection of clinically relevant genetic variants

**DOI:** 10.3389/fonc.2022.1014592

**Published:** 2022-12-27

**Authors:** Ariane Hallermayr, Teresa M. Neuhann, Verena Steinke-Lange, Florentine Scharf, Andreas Laner, Roland Ewald, Ben Liesfeld, Elke Holinski-Feder, Julia M. A. Pickl

**Affiliations:** ^1^ MGZ – Medizinisch Genetisches Zentrum, Munich, Germany; ^2^ Pettenkofer School of Public Health, Munich, Germany; ^3^ Institute for Medical Information Processing, Biometry, and Epidemiology –IBE, Ludwig-Maximilians-Universität (LMU) Munich, Munich, Germany; ^4^ Medizinische Klinik und Poliklinik IV, Klinikum der Universität München, Munich, Germany; ^5^ Limbus Medical Technologies GmbH, Rostock, Germany

**Keywords:** liquid biopsy, tissue biopsy, circulating tumor DNA, analytical validation, Duplex sequencing, cancer, asymmetric overgrowth syndrome

## Abstract

**Background:**

Liquid biopsy (LB) is a promising complement to tissue biopsy for detection of clinically relevant genetic variants in cancer and mosaic diseases. A combined workflow to enable parallel tissue and LB analysis is required to maximize diagnostic yield for patients.

**Methods:**

We developed and validated a cost-efficient combined next-generation sequencing (NGS) workflow for both tissue and LB samples, and applied Duplex sequencing technology for highly accurate detection of low frequency variants in plasma. Clinically relevant cutoffs for variant reporting and quantification were established.

**Results:**

We investigated assay performance characteristics for very low amounts of clinically relevant variants. In plasma, the assay achieved 100% sensitivity and 92.3% positive predictive value (PPV) for single nucleotide variants (SNVs) and 91.7% sensitivity and 100% PPV for insertions and deletions (InDel) in clinically relevant hotspots with 0.5-5% variant allele frequencies (VAFs). We further established a cutoff for reporting variants (i.e. Limit of Blank, LOB) at 0.25% VAF and a cutoff for quantification (i.e. Limit of Quantification, LOQ) at 5% VAF in plasma for accurate clinical interpretation of analysis results. With our LB approach, we were able to identify the molecular cause of a clinically confirmed asymmetric overgrowth syndrome in a 10-year old child that would have remained undetected with tissue analysis as well as other molecular diagnostic approaches.

**Conclusion:**

Our flexible and cost-efficient workflow allows analysis of both tissue and LB samples and provides clinically relevant cutoffs for variant reporting and precise quantification. Complementing tissue analysis by LB is likely to increase diagnostic yield for patients with molecular diseases.

## 1 Introduction

LB enables identification of genetic sequence variants in circulating free DNA (cfDNA) from plasma and allows stratification of patients that will benefit from targeted therapies. One example is the detection of oncogenic driver variants in plasma of non-small cell lung cancer (NSCLC) patients that is associated with response to tyrosine kinase inhibitor therapy (1).

LB is an alternative to standard tissue biopsy, and is increasingly applied when tissue accessibility is limited, in case tissue biopsy leads to insufficient quality or quantity of material, or the result of a tissue biopsy analysis is expected to take longer than a LB assay (2). Accordingly, LB has been acknowledged as possible alternative to tissue biopsy by U.S. and European guidelines and position papers (3–6). With ~16,000 advanced NSCLC patients per year in Europe who are eligible for biomarker analysis but do not receive biomarker testing due to unsuccessful tissue biopsy, there is a huge potential of LB to increase the number of patients that benefit from personalized treatment (2). These include not only NSCLC or other solid tumor patients, but also patients with mosaic diseases.

A major advantage of LB compared to tissue biopsy is that LB is able to cover the genetic heterogeneity of disease (7–9). Cells carrying the disease-causing variant release their cfDNA into circulation, which can then be analyzed using LB. In contrast, these cells may be missed by tissue analysis, as it is commonly performed at one site at a singular time point. Accordingly, addition of LB to tissue biopsy has shown to add significant diagnostic value (1). Nevertheless, LB has not widely been implemented into routine clinical practice to date.

Aiming at offering LB to as many patients as possible, clinical laboratories must 1) cover the important therapy relevant variants, 2) use validated highly sensitive and accurate methods for detection of very low variant frequencies in plasma with well-defined cutoffs for variant reporting and quantification, and 3) offer both complementary and combined analysis of plasma and tissue biopsies for full flexibility and to maximize diagnostic yield.

The aim of this study is to develop aNGS-based assay that combines liquid and tissue biopsy (including tumor tissue and fibroblasts) to maximize flexibility for both patients and clinicians and to increase diagnostic yield at acceptable cost. We selected the most important therapy relevant variants and considered latest technological improvements for detection of very low frequency variants commonly present in plasma (3, 5, 10–14). We performed analytical validation of our workflows including definition of cutoffs for variant reporting and quantification, and describe a clinical case where LB was able to provide molecular diagnosis of overgrowth syndrome that could not be identified by tissue biopsy and other molecular analyses approaches.

## 2 Materials and methods

### 2.1 Ethics approval and consent to participate

The study was approved by the ethics commission of the Bavarian Medical Association (No. 17059) and is registered with the German registry for clinical trials (trial registration ID: DRKS00012890). All participants or their legal guardian provided informed written consent prior to blood and tissue specimen collection. For the case report, the legal guardian provided written consent for publication of pictures. The study was performed in accordance with the Declaration of Helsinki.

### 2.2 Patient samples

Skin fibroblast and plasma samples were obtained in parallel from a ten-year old girl with clinically diagnosed asymmetric overgrowth syndrome. Another plasma sample was obtained from one tumor patient and FFPE tissue samples were collected from a total of 12 tumor patients (**Supplementary Table 1**).

### 2.3 Reference materials, DNA extraction, kit design, library preparation, karyotyping, microarray analysis and whole-exome sequencing

Information on reference materials, DNA extraction, kit design, library preparation and sequencing, karyotyping, microarray analysis, and whole-exome sequencing (WES) are provided in the **Supplementary Methods**.

### 2.4 Bioinformatics analysis

Raw data (FASTQ.GZ format) was uploaded to the VARVIS^®^ platform and aligned against the hg38 reference genome followed by variant calling using the bioinformatics pipeline VARFEED worker 1.5.1 with *in silico* validated standard settings. Within the LB workflow the Duplex consensus was built by extraction and processing of Duplex sequencing barcodes according to Schmitt et al. (14) and the manufacturer's "analysis guidelines, version 1" (15). A minimum of two reads were used to construct a strand-specific consensus read. Strand-specific consensus reads were then combined to create a final consensus read. No consensus was built within the tissue analysis workflow.

### 2.5 Validation of Duplex sequencing

#### 2.5.1 Validation samples

To allow 95% confidence for detection of variants present with low VAFs, we aimed to analyze at least 60 low frequency variants with both LB and tissue analysis (16–18). The LB analysis was validated using the Seraseq^®^ ctDNA Complete™ Reference Materials (LGC seracare) with 0%, 0.05%, 0.1%, 0.5%, 1% and 5% VAF. Performance was evaluated based on the single well-characterized genomic background (GM24385) (19) including 59 SNVs and 1 InDel, and spike-in variants present in the respective VAF including 8 SNVs and 7 InDels. The analysis of the wild type (WT) once and each of the spike-in reference materials in duplicates covered a total of 660 germline variants (649 SNVs, 11 InDels) and 90 spike-in variants present in VAFs above the LOB (48 SNVs, 42 InDels).

Validation of the tissue analysis was performed using the Quantitative Multiplex Reference Standard FFPE (Horizon), ten well-characterized clinical samples and the Ashkenazim Son FFPE Reference Standard NA24385 (SensID) as WT control. Further, *in silico* dilutions of three of the well-characterized clinical samples in the Ashkenazim Son FFPE Reference Standard NA24385 (SensID) were generated with 8% to 16% VAF of spike-in variants. Performance was evaluated based on a total of 573 somatic variants (546 SNVs, 27 InDels).

#### 2.5.2 Determination of the LOB

To limit the number of false positive (FP) variants, we established the LOB at 0.25% VAF based on the background noise at genomic positions expected to be WT in all samples as cutoff for variant calling. With a target region of 102 kb the number of positions in each of the validation samples for both LB and tissue analysis exceeds the required 60 WT positions for determination of the LOB (16). Further, we established that variants are expected to be true positives (TP), when they are present in at least eight consensus reads.

#### 2.5.3 Determination of the limit of detection

For determination of the LOD we investigated the detection rate of 90 spike-in variants from 0.5% to 5% VAF for LB analysis and of 573 variants with 8% to 16% VAF for tissue analysis. The number of analyzed spike-in variants exceeds the required 60 variants for determination of the LOD with 95% confidence (16, 17). Since we detected 27/30 variants with 0.5% VAF the LOD could be established at 0.5% VAF with 90% confidence. All missed variants were InDels, therefore the performance for InDel detection is expected to be significantly lower than for SNVs.

#### 2.5.4 Determination of sensitivity and PPV

Variants were detected above the LOB of the respective analysis (LB: 0.25%, tissue: 5%) as cutoff for true-positive variant detection. The variants detected in processed samples were compared to the intersected trusted regions of the GIAB version 3.3.2 (19) reference data set using vcfeval (Real Time Genomics) (20). Subsequently, sensitivity and PPV were separately assessed for SNVs and InDels. The number of InDels analyzed with both LB and tissue analysis is very low and thus does not yield accurate information. However, as described above, the performance for InDel detection is expected to be significantly lower than for SNVs.

#### 2.5.5 Determination of the LOQ

In addition to sensitivity and PPV, we also assessed trueness, precision and the total error of 30 spike-in variants with 0.5% VAF, 1% VAF and 5% VAF, respectively, to establish the LOQ (**Supplementary Methods**). Guidelines recommend analyzing 40 variants of the target VAF to establish the LOQ (16), however with 30 variants at the respective VAF we were able to estimate the total error, required for determination of the LOQ.

## 3 Results

### 3.1 Assay design

Our aim was to develop a cost-efficient workflow for both LB and tissue analysis which covers the most important actionable genes and variants in solid tumors and mosaic diseases based on a custom hybrid-capture panel for targeted sequencing on the basis of European Society for Medical Oncology (ESMO) guidelines (3, 5, 11–13) and current clinical trials (21–27) (i.e. 30 genes and hotspots of three additional genes, **Supplementary Methods**, **Supplementary Table 2**). Our final panel targets in total 102 kb, and includes 500 exons of canonical and 33 exons of non-canonical transcripts. According to a comprehensive catalogue of hotspot variants identified in ~25.000 tumor samples (28) our assay interrogates 370 known variants in the target region (**Supplementary Table 4**).

### 3.2 Workflow design

**Figure 1:**
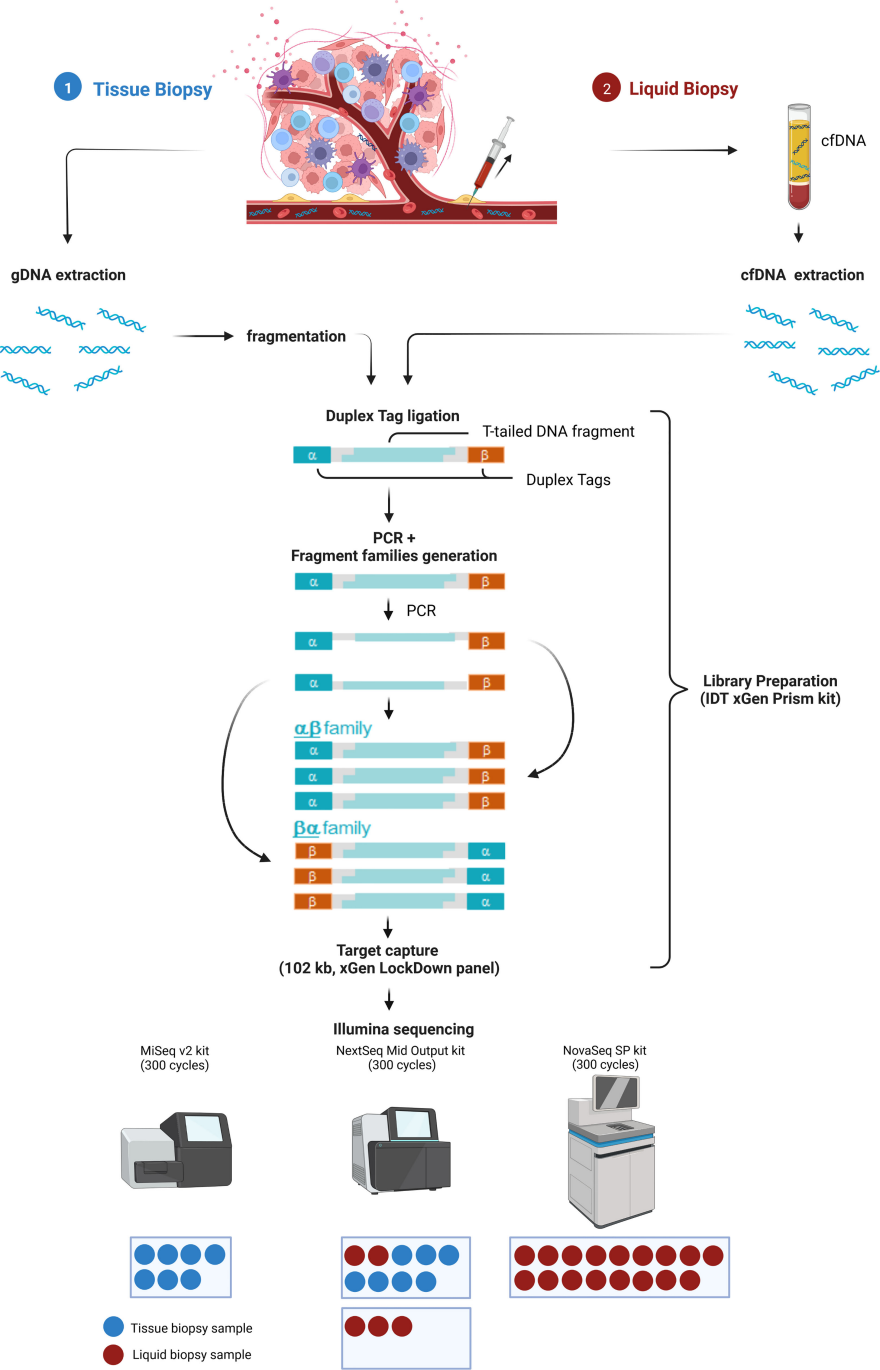
Combined analysis workflow for plasma and tissue samples. Created with BioRender.com.

To allow maximal flexibility we aimed at enabling plasma and tissue sample processing either in parallel or independently (**Figure 1**). After genomic DNA (gDNA) extraction from tissue followed by fragmentation and cfDNA extraction from plasma, both sample types are processed together beginning with library preparation, including the ligation of strand-aware barcodes (Duplex tags) for highly sensitive variant detection (29). While the combined workflow ensures a similar turnaround time, the additional fragmentation required for tissue analysis results in a slightly shorter turnaround time for LB analysis. To achieve ~90 Mio and ~4 Mio raw reads of plasma and tissue samples, respectively, target capture is performed in separate pre-pools for plasma and tissue samples followed by pooling in the appropriate ratio for sequencing. Depending on the number of plasma and tissue samples, various sequencing options are possible (**Figure 1**).

### 3.3 Consensus building for LB analysis

**Figure 2:**
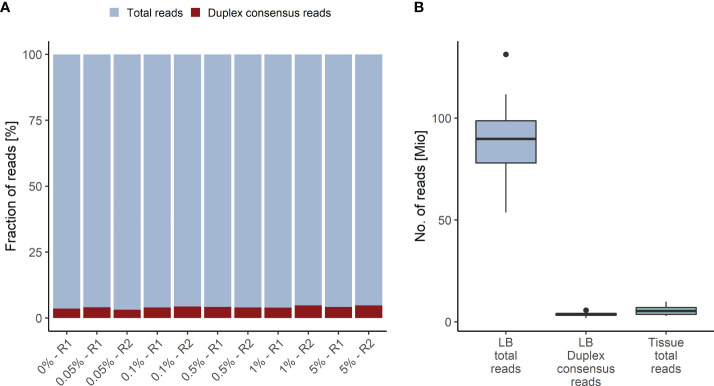
Duplex Consensus building. **(A)** Duplex consensus reads (red) as fraction of total reads (blue). **(B)** Total reads and Duplex consensus of plasma samples vs. total reads of tissue samples. R1, replicate 1; R2, replicate 2.

The Duplex tags added during library preparation allow the bioinformatics identification of all amplified DNA fragments originating from a single strand of the original DNA molecule (29). PCR amplified fragments originating from the forward strand are assigned to the αβ fragment family, whereas PCR amplicons from the complementary strand are assigned to the βα fragment family (**Figure 1**). Each fragment family builds a single strand consensus to filter out false positives including sequencing errors and late PCR artifacts (**Supplementary Figure 1**). For LB analysis both strands are combined to build the Duplex consensus (29), which enables the detection of variants frequently present in very low VAFs in plasma by reducing the background noise (30). In our validation samples with a median ~89.8 million total reads were collapsed to a median of ~3.6 million Duplex consensus reads, leading to ~95.9% consensus reduction (**Figure 2A**; **Supplementary Table 5**). In contrast, for tissue samples a median of ~5.4 million total reads were achieved, for which no consensus was built (**Figure 2B**; **Supplementary Table 5**).

With our approach, we achieved a mean effective coverage of 1,735x and 1,496x for plasma and tissue samples, respectively. For plasma samples in median ~98.6% of regions were covered with >250x, and for tissue samples ~100% of regions were covered with >100x (**Supplementary Table 6**).

### 3.4 Limit of Blank definition and variant detection rates

**Figure 3:**
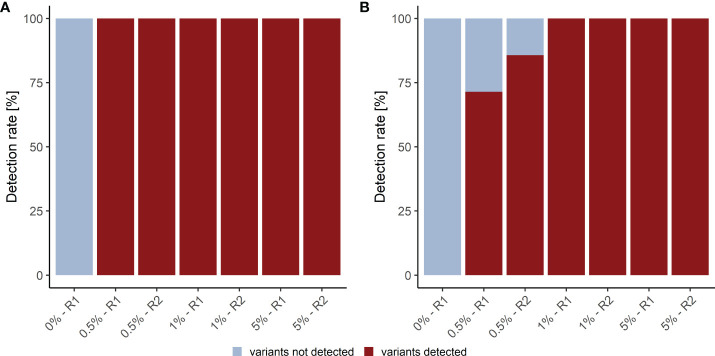
Detection rate of plasma analysis. **(A)** SNV and **(B)** InDel detection rate in plasma reference samples. R1, replicate 1; R2, replicate 2.

We assessed the detection rate in plasma samples using cfDNA isolated from Seraseq^®^ ctDNA Complete™ Reference Materials (VAF 0%-5%) (Seracare). The Seraseq^®^ ctDNA Complete™ Reference Material includes 40 clinically relevant variants across 28 genes at 0.5%, 1% and 5% and a WT control sample. Of these variants 15 (8 SNVs and 7 InDels) are located in the kit target region. Only variants with a VAF above the LOB of 0.25% for the LB Duplex workflow that are present in at least eight consensus reads were called. Using these parameters, we observed a SNV detection rate of 100% at 0.5%, 1.0% and 5.0% VAF and an InDel detection rate of 79% at 0.5% VAF (average of duplicate measurement), and of 100% at 1% and 5% VAF, with highly similar detection in forward and reverse reads (**Figure 3**; **Supplementary Table 7**). Notably, neither SNVs nor InDels contained in the reference material were detected in the WT sample.

We further analyzed the detection rate in tissue samples using gDNA isolated from Quantitative Multiplex Reference Standard FFPE (Horizon), ten well-characterized clinical samples and the Ashkenazim Son FFPE Reference Standard NA24385 (SensID) as WT control. 7/11 clinically relevant variants of the Quantitative Multiplex Reference Standard FFPE (Horizon) and 11/11 clinically relevant variants in ten clinical samples were covered in the kit target region and above the LOB of 5% for the tissue analysis workflow. For tissue samples, a detection rate of 100% was observed since all 18 variants present in different reference materials with VAFs above the LOB of 5% were detected. As expected, no clinically relevant variants were detected in the Ashkenazim Son FFPE Reference Standard NA24385 (SensID) WT control (**Supplementary Table 7**).

### 3.5 Sensitivity and PPV of plasma samples

To determine the sensitivity of LB analysis, we considered TP in all reference materials with 0.5%, 1.0% and 5.0% VAF. In total, 48/48 SNVs and 39/42 InDels were detected, resulting in 100% and 92.9% sensitivity for SNVs and InDels, respectively (**Supplementary Table 7**). In addition to sensitivity, we also determined the PPV considering TPs and FPs. Thereby, the PPV of the LB analysis was established to be 63.2% (48/76) for SNVs and 84.8% (39/46) for InDels (**Table 1**; **Supplementary Table 7**). One possible reason for the high number of FPs is the poor characterization of the germline background of the reference materials (19). Consequently, it is difficult to assess whether all FPs are indeed FPs or possibly TPs. To limit this issue, we further focused on the 2.5 kb of our target region overlapping with hotspot regions identified in 24.592 tumors reported by Chang et al. (28) (**Supplementary Table 9**). A total of 48/48 SNVs and 33/36 InDels with VAFs from 0.5%-5% were detected as TP variants, resulting in 100% and 91.7% sensitivity, respectively. Further, a PPV of 92.3% (48/52) for SNVs and of 100% (33/33) for InDels could be established in these hotspot regions (**Table 1**).

**Table 1:** Sensitivity and PPV of LB and tissue analyses.

LB analysis	
**Complete target region (102 kb)**	
*SNVs*	
*TP*	48
*FN*	0
*FP*	28
***Sensitivity***	**100.00%**
***PPV***	**63.20%**
*InDels*	
*TP*	39
*FN*	3
*FP*	7
***Sensitivity***	**92.90%**
***PPV***	**84.80%**
**Cancer hotspot regions (2.5 kb)**	
*SNVs*	
*TP*	48
*FN*	0
*FP*	4
***Sensitivity***	**100.00%**
***PPV***	**92.30%**
*InDels*	
*TP*	33
*FN*	3
*FP*	0
***Sensitivity***	**91.70%**
***PPV***	**100.00%**
***Tissue analysis***	
*SNVs*	
*TP*	515
*FN*	3
*FP*	0
***Sensitivity***	**99.40%**
***PPV***	**100.00%**
*InDels*	
*TP*	22
*FN*	0
*FP*	3
***Sensitivity***	**100.00%**
***PPV***	**88.00%**

PPV and Sensitivity are calculated based on TP, FN, and FP results.

TP, true positives; FN, false negatives; FP, false positives; PPV, positive predictive value.

### 3.6 Sensitivity and PPV of tissue samples

To evaluate sensitivity and PPV of the tissue analysis, *in silico* dilutions of 10% and 20% of three internal reference materials (T-CRC-04, T-EC-01, and T-NF-01), previously characterized by WES, were generated in the Ashkenazim Son FFPE Reference Standard NA24385 (SensID) as well characterized background (19). Depending on the previously determined VAF of individual variants, present in the three internal reference materials, the generated *in silico* dilutions were expected to contain spike-in variants in the range from 8% to 16% VAF. For SNVs 99.4% (512/518) sensitivity and 100% (515/515) PPV was achieved. For InDels, 100% (22/22) sensitivity and 88% (22/25) PPV was obtained (**Table 1**).

### 3.7 Limit of quantification

To enable disease monitoring in cancer patients using LB, we established the LOQ for the LB Duplex sequencing workflow. The LOQ represents the cutoff above which VAFs can be accurately quantified based on acceptable trueness (>90%) and precision (>80%) (**Supplementary Methods**).

To estimate trueness, representing the closeness of agreement between measured and reference VAF, we calculated the bias between actual VAFs (confirmed by digital droplet PCR) and measured VAFs of variants present in each plasma reference material. Variants with ~0.5% VAF were determined with 85.0% trueness, variants with ~1% VAF with trueness of 92.5%, and variants with ~5% VAF with 99.9% trueness (**Supplementary Figure 2A**; **Supplementary Table 10**). Therefore, the goal for trueness was achieved with VAFs from 1%.

We further established precision in terms of repeatability based on each variant present in reference materials. Therefore, we calculated the pooled standard deviation over all variants between the two replicates of each reference material. For variants with ~0.5% VAF, ~1% and ~5% VAF repeatability was determined to be 34.4%, 65.2%, and 91.9%, respectively (**Supplementary Figure 2B**, **Supplementary Table 10**). Therefore, the goal for precision was achieved with VAFs from 5%.

Based on these results we were able to establish the LOQ as cutoff for VAF quantification at a VAF of 5% with a total error of 16.2%. These results indicate that variants determined with VAFs ≥5% can be reliably quantified with our LB Duplex sequencing workflow and therefore are informative for disease monitoring. Since tissue analysis cannot be used for disease monitoring, we only validated the tissue analysis with qualitative rather than quantitative intent. However, measured VAFs in two replicates for most of the variants deviated only 0.2% to 7.9%. Only for the *PIK3CA* (NM_006218.4) p.E545K, a VAF of ~34.2% higher than the true VAF of 8.3% was observed.

### 3.8 LB increases diagnostic yield by identifying the molecular cause of disease

**Figure 4:**
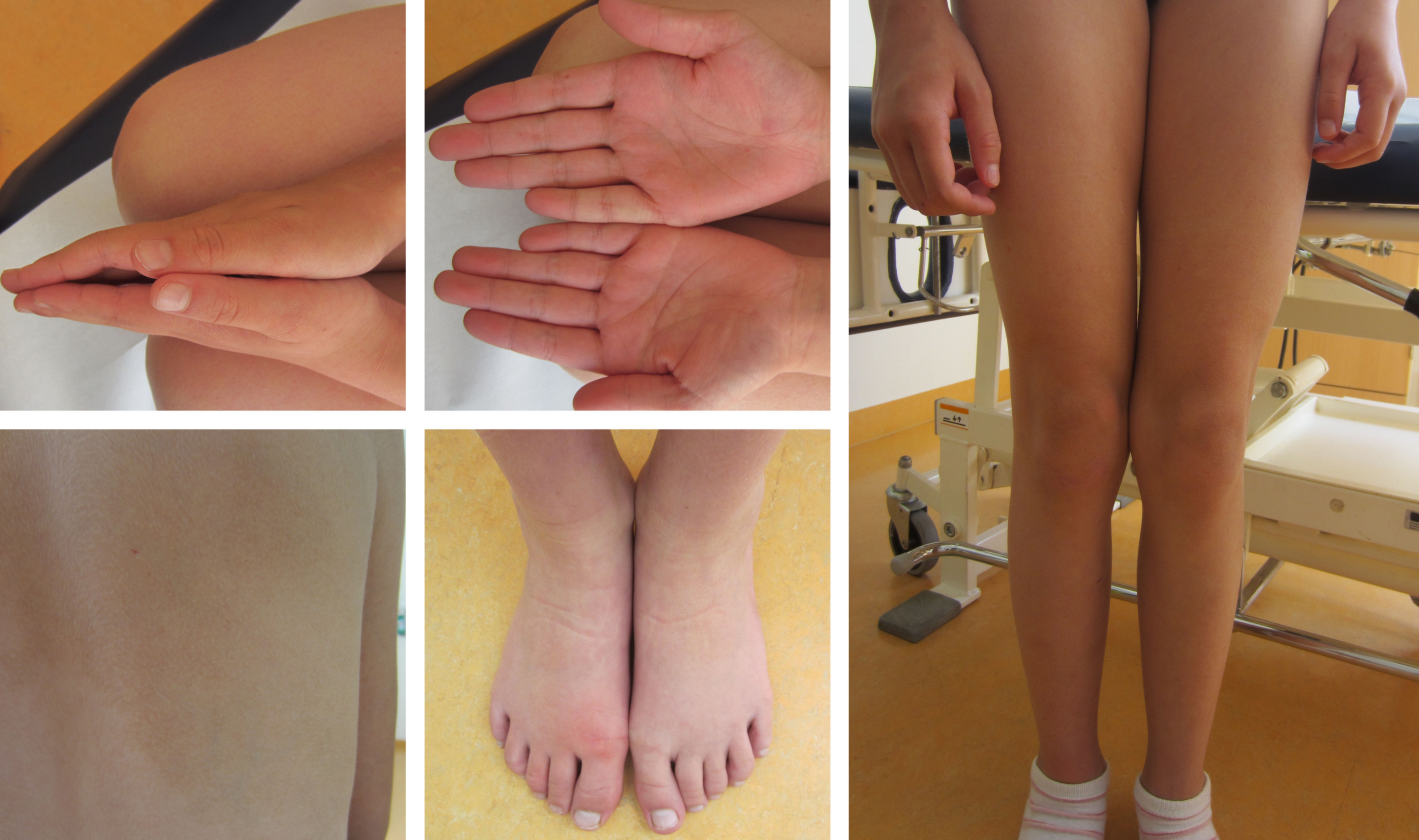
Clinically confirmed overgrowth syndrome in a 10-year-old girl to be analyzed for molecular clarification. Marked asymmetry of hands and legs. The feet have a difference in circumference. On the back, there is a large asymmetric reddish-brown area of skin, with temperature-depended hyperpigmentation, suggestive of capillary malformations in the area.

To test the diagnostic value of our approach, we applied plasma and tissue analysis with our combined workflow in samples of a ten-year old girl with clinically diagnosed asymmetric overgrowth syndrome including arteriovenous malformations in the right arm and right leg, but without molecular diagnosis that would support personalized treatment (**Figure 4**). For diagnosis of this phenotype standard procedure is a skin biopsy to identify mosaicism of pathogenic variants in associated genes (31). Since skin fibroblasts do not necessarily harbor the disease-causing variant in patients with vascular phenotypes, but the affected vascular tissue releases DNA into circulation, LB can be informative as well for the molecular characterization of disease.

Initially, standard molecular diagnostics were performed including karyotyping from heparin blood, which provided inconspicuous results and microarray analysis from EDTA blood that identified a duplication of 15q13.3 not related to the phenotype. The following WES from skin fibroblasts did not identify any disease-causing variant. Using our tissue analysis workflow including 17 genes associated with overgrowth syndrome, also no pathogenic variant could be identified, whereas LB analysis identified *KRAS* (NM_004985.5) c.35G>A p.(Gly12Asp) in plasma with a measured VAF of 1.01% (**Table 2**). The *KRAS* c.35G>A, p.(Gly12Asp) variant leads to constitutive overactivation and increased signal transduction into downstream pathways and is associated with overgrowth including various types of congenital nevi and vascular malformations (so-called mosaic RASopathies) (32, 33). Our result is in line with a previous study identifying this variant as the molecular cause of asymmetric overgrowth syndrome and vascular malformation (33). Because all available cfDNA from the patient was used as input for LB analysis, the detected variant could not be verified using an orthogonal method such as droplet digital PCR (ddPCR). However, the detection of this variant in accordance with the previously validated performance criteria and the perfect genotype-phenotype correlation in combination with previous findings describing the respective variant as the cause of asymmetric overgrowth syndrome and vascular malformation suggest that this variant is a true positive. Taken together, detection of *KRAS* c.35G>A, p.(Gly12Asp) variant using LB that could not be identified with the tissue workflow, molecularly explained the clinically diagnosed overgrowth syndrome in our patient, hereby providing personalized treatment options including MEK inhibitors.

**Table 2:** Molecular analysis of clinically confirmed overgrowth syndrome.

Method	Result	Variant	Measured VAF	Material	LOD
*Karyotyping*	unsuspicious	–	–	Heparin blood	
*Microarray*	VUS (CNV - not related to phenotype)	Duplication of 15q13.3	–	EDTA blood	
*WES^a^*	unsuspicious	–	–	Skin fibroblasts	20%
*Tissue analysis^b^*	unsuspicious	–	–	Skin fibroblasts	8%
*LB analysis^b^*	SNV pathogenic	***KRAS* c.35G>A p.(Gly12Asp)**	**1.01%**	Plasma	0.5%

Analyzed genes: ^a^AKT1, AKT3, GNAQ, GNAS, IDH1, IDH2, IKBKG, KRAS, MTOR, NF1, NRAS, NSDHL, PIK3CA, PIK3R2, PORCN, PTCH1. PTEN, RASA1, SPRED1, TSC1, TSC2; ^b^HRAS, FGFR1, KRAS, AKT3, BRAF, MTOR, CCND2, GNA11, GNAQ, NRAS, MAP2K1, RASA1, EPHB4, PIK3R2, SMO, PIK3CA, GNAS;

## 4 Discussion

LB is a promising tool in precision medicine. It is fast, non-invasive and represents disease heterogeneity at any desired time point (34–36). Several institutions including the National Comprehensive Cancer Network (NCCN), the College of American Pathologists (CAP), the International Association for the Study of Lung Cancer (IASLC), the Association for Molecular Pathology (AMP), the ESMO and the ESMO Precision Medicine Working Group (PMWG) have acknowledged the advantage of LB for patient management (3–6, 37). On national level, LB testing has already been included in clinical guidelines, and health insurances have started to reimburse LB analysis. Besides its high costs, a major challenge of LB for its usage in clinical practice, however, is the reliable detection with defined cutoffs for variant reporting and quantification of low variant allele frequencies in plasma that are especially common in patients with non-metastatic cancer and mosaic diseases (30). Here we developed a flexible workflow for parallel analysis of plasma and tissue samples to maximize diagnostic yield and provide LOB and LOQ as clinically relevant VAF reporting and quantification cutoffs.

A major challenge for implementation of highly sensitive LB analysis into clinical practice are the generally high costs due to the high sequencing coverage required. In our workflow, one to two LB samples can be pooled with seven tissue samples on an Illumina NextSeq flowcell (Mid output), at reasonable cost. Notably, combining 17 plasma samples on a NovaSeq SP flowcell (i.e. the maximal number of LB samples that can be pooled on this flowcell) reduces costs per sample significantly, which is in the range of targeted LB hotspot analysis using digital droplet PCR and tissue sample analysis. Notably, adding the option of LB analysis to clinical labs not only results in higher flexibility but also reduces costs for tissue sample analyses. Taken together, to the best of our knowledge this is the most cost-effective approach for a mid-size and highly sensitive NGS LB panel.

Using our workflow, LB samples can be processed in parallel to tissue samples similar to the MSI-ACCESS assay developed by the Memorial Sloan Kettering Cancer Center (MSK), processing LB samples from cancer patients in parallel to white blood cells (WBC) (38). This assay is also based on LB Duplex sequencing and achieved a slightly lower sensitivity for both hotspot and *de novo* variants, but with a higher PPV. Comparing performance characteristics of our workflow to five other commonly used LB SNV detection assays tested with the same reference material in Weber et al. (39), our assay was the only one with all SNVs detected at 0.5% VAF and none in the WT control, indicating a low number of false positives. Further, our 102 kb target region encompassed mainly the whole coding sequence of targeted genes, whereas the five investigated assays primarily focused on hotspot regions. The only assay targeting the whole coding sequence was the Oncomine Lung cfDNA assay (Thermo Fisher), encompassing 12 genes associated with lung cancer, but thereby restricting its application to a smaller patient population. This assay was also the best performing one, showing similar sensitivity and PPV values to our approach. However, this assay relies on the Ion Torrent platform, whereas our assay uses the Illumina platform, which enables broader application as it is more widespread. Further, our approach is the only one combining the analysis of LB and tissue samples for the detection of both tumor and mosaic diseases, making it an easily implementable workflow in clinical practice.

Rather than only including actionable variants for mosaic disease, we show with our case report a clinical proof-of-concept that LB can be extended from cancers to all heterogeneous diseases such as mosaic diseases, including asymmetric overgrowth syndromes (e.g. Proteus syndrome, Klippel-Trennaunay syndrome and *PIK3CA*-related overgrowth spectrum, PROS, as well as many others). Detection of somatic variants in cancer and PROS can guide personalized treatment including consideration of inhibitors of the PI3K/AKT/mTOR signaling pathway. Notably, simple blood draws required for LB are much more convenient for patients than tissue biopsies, especially for children, which are frequently affected by mosaic diseases.

However, there are limitations that need to be considered. Accurate detection of variants <0.5% VAF is challenging. This finding is in line with a recent study testing five leading commercial ctDNA assays, which show generally high performance for VAFs ≥0.5% (40). Accordingly, the main application is molecular stratification and profiling of tumor evolution in patients with advanced cancers, where variants are commonly detected with VAFs ranging from 1% to 10% (40). Another limitation represents the small sample size for the analytical validation of the LB analysis. However, there are only a limited number of reference materials with well-defined low-frequency variants available. Using the Seraseq^®^ ctDNA Complete™ Reference Materials with different VAFs of spike-in enabled accurate validation of our approach based on a total of 90 spike-in variants and 660 germline variants. We further showed a clinical proof-of-concept for application of LB analysis for genotyping of mosaic disease in only one case. To show clinical validity in addition to analytical validity of LB analysis in patients with mosaic disease LB analysis in parallel to clinical evaluation in more patients would be required. Furthermore, the LB analysis was initially analytically validated by establishing the LOB as cutoff for variant detection and the LOQ as cutoff for variant quantification. However, it was not clinically validated for residual disease detection or treatment monitoring in cancer patients. To apply LB analysis also for residual disease detection based on the LOB or treatment monitoring by tracking changes in VAF above the LOQ, its prognostic value needs to be evaluated in an independent study using follow-up samples from patients with known somatic variants. Nevertheless, as described in previous studies, it is likely that the presence of post-surgery ctDNA is likely to indicate residual disease, and changes in VAFs throughout treatment are likely to indicate response or resistance (41–43). To determine clinical utility of LB analysis for treatment monitoring prospective studies would be required guiding treatment decisions based on presence of post-surgery ctDNA or changes in VAFs.

In conclusion, LB is capable to detect the complete mutational profile of both the primary tumor and metastatic lesions. LB Duplex sequencing pushes the boundaries for detection of low frequency variants in plasma with NGS based analysis. Our broad Duplex sequencing panel enables highly sensitive detection of therapy relevant variants in tumor and mosaic diseases. We were able to identify the *KRAS* c.35G>A, p.(Gly12Asp) variant in plasma as the molecular cause of the clinically confirmed overgrowth syndrome in a ten-year old girl, which could not be detected in the analysis of skin fibroblasts using our tissue workflow, which may be due to heterogeneity not depicted in the resected specimen. The identification of the *KRAS* variant may lead to novel therapy options, highlighting the diagnostic value of LB analysis for heterogeneous diseases in clinical practice. In summary, our workflow that easily combines tissue and LB analysis has the potential to increase the diagnostic yield, which is in line with previous results, identifying an increase of diagnostic yield by ~15% due to introduction of LB as tissue analysis alternative (1).

## Data availability statement

The sequence data have been deposited at the European Genome-phenome Archive (EGA) under accession number EGAS00001006805. The original contributions presented in the study are included in the article/**Supplementary Material**. Further inquiries can be directed to the corresponding author.

## Ethics statement

The studies involving human participants were reviewed and approved by ethics commission of the Bavarian Medical Association. Written informed consent to participate in this study was provided by the participants' legal guardian/next of kin. Written informed consent was obtained from the minor(s)' legal guardian/next of kin for the publication of any potentially identifiable images or data included in this article.

## Author contributions

TN, VS-L, and JP researched the literature to identify cancer and PROS associated genes. FS established the target region of the sequencing panel. AH developed the experimental procedures, and performed all experiments. AH, AL, RE, BL, and JP analyzed and interpreted the data. AH, RE, and BL performed statistical analysis. AH and JP designed the study. JP supervised the work. EH-F provided financial and technical resources to enable conduction of the study. AH and JP wrote the manuscript. All authors contributed to the article and approved the submitted version.
