# Supplementary material for: Highly sensitive liquid biopsy Duplex sequencing complements tissue biopsy to enhance detection of clinically relevant genetic variants
Source: Front Oncol. 2022 Dec 27;12:1014592. doi: 10.3389/fonc.2022.1014592 (PMC9831673; doi:10.3389/fonc.2022.1014592)
Supplement: Supplementary file 1 [file DataSheet_1.pdf]

Supplementary Material

1 Supplementary Methods

1.1 Reference materials

For validation of the plasma analysis, cfDNA isolated from Seraseq® ctDNA Complete™ Reference Material (LGC seracare, Milford, Massachusetts, USA) with spike-in variant allele frequencies (VAFs) ranging from 0.1% to 5% (8 SNVs, 7 InDels) (#Cat 0710-0669, 0710-0671, 0710-0672, 0710-0673) in a single well-characterized genomic background (GM24385) (1) (59 SNVs, 1 InDel) and the respective WT control (Cat 0710-0674) were used (Supplementary Table 1). Further, an additional control with 0.05% VAF was generated by spiking cfDNA from the 0.1% VAF reference material in cfDNA from the WT reference material.

Genomic DNA (gDNA) isolated from formalin-fixed paraffin embedded (FFPE) tissue of the Quantitative Multiplex Reference Standard (FFPE) (Horizon Discovery Ltd, #Cat HD200), the Ashkenazim Son FFPE Reference Standard NA24385 (SensID, #Cat SID-000100), and eleven internal controls previously analyzed with WES were used for validation of the tissue analysis workflow (16 SNVs, 5 InDels) (Supplementary Table 1). *In silico* dilutions with 10% and 20% of three of the internal controls in the Ashkenazim Son FFPE Reference Standard NA24385 (SensID) were generated with target VAFs of 8% to 16% of spike-in variants (518 SNVs, 22 InDels).

1.2 Extraction of cfDNA

CfDNA from three to 5 ml plasma was isolated using the QIAamp circulating nucleic acid kit (Qiagen, #Cat 55114) according to manufacturer's instructions. All buffer volumes were adjusted to the respective plasma volumes. All membrane washing steps were performed twice. CfDNA concentration was quantified using the High Sensitivity NGS Fragment Analysis Kit (Agilent, #DNF-474-0500) on the Fragment Analyzer system (Agilent).

1.3 Extraction of gDNA from tumor tissue and skin fibroblasts

Genomic DNA from FFPE tissue was isolated using the QIAamp DNA FFPE Tissue Kit (Qiagen, #Cat 56404) as by manufacturer's instructions. gDNA from skin fibroblasts was isolated using the FlexiGene DNA Kit (Qiagen, #Cat 51206) as by manufacturer's instructions. gDNA from whole blood was extracted on a Biomek® FX system (Beckman Coulter, Brea, California, USA) using the NucleoMag® Blood 3 ml Kit (Machery-Nagel, #REF 744502.1) as by manufacturer's instructions. Concentration of gDNA was determined using a High Lunatic Plate (Strips D+) (#Cat 7012004) on a Lunatic system (Unchained Labs).

1.4 Kit design

The target region of our Duplex sequencing approach comprises the coding region + 5 bp at exon-intron borders of 30 genes and hotspots of three additional genes associated with cancer and asymmetric overgrowth syndromes (e.g. Proteus syndrome, Klippel-Trennaunay syndrome, PIK3CA-

related overgrowth syndrome / PROS, etc.) leading to a target region of 102 kb. In addition, 16 common SNPs for identity control were included in the target region (Supplemental Table 1). A custom xGen Lockdown Probe Pool (Integrated DNA Technologies, Inc.; IDT) was created based on this target region (Supplemental Table 2).

1.5 Library preparation and sequencing

Library preparation of cfDNA and gDNA samples was performed using the xGen Prism DNA Library Prep Kit (IDT, #Cat 10006203) as by manufacturers' instructions. 10 to 25 ng of cfDNA and 25 to 100 ng of gDNA were used as input material for library preparation. Fragmentation of gDNA from FFPE tissue and skin fibroblasts was performed with a pulsed shearing program on the Covaris E220 system (Covaris, LLC.) in 8 microTUBE-50 AFA Fiber Strip V2 (Covaris, #Cat 520174) with the following settings: 18 repeats of 10 sec treatment with a peak incident power of 75 W, 15% duty factor, and 500 cycles per burst at 7°C. Libraries from cfDNA and gDNA were amplified with nine PCR cycles. Libraries were quantified using a DNA 1000 Kit (Agilent, #Cat 5067-1505) on the Bioanalyzer system (Agilent). Following library preparation target enrichment was performed in accordance with the xGen hybrid-capture of DNA libraries protocol (IDT, v4, May 2019) using the xGen Lockdown Probe Pools (Integrated DNA Technologies, as described in Kit design), the xGen Hybridization and Wash Kit (IDT, #Cat 1080577), and the xGen Library Amplification Primer Mix (IDT, #Cat 1077675). Prior to hybridization, two to three cfDNA samples or seven gDNA samples were pooled equimolarly. Hybridization was performed over 14 to 16 h and final libraries were amplified with 11 PCR cycles. Pre-pools were quantified using a High Sensitivity DNA Kit (Agilent, #Cat 5067-4626) on the Bioanalyzer system (Agilent). Paired-end sequencing with 2x151 bp reads was performed on a MiSeq or NextSeq 500 system (Illumina). CfDNA pre-pools of three samples were sequenced on a NextSeq Mid output flowcell, and gDNA pre-pools of seven samples either were sequenced on the MiSeq system or pooled with cfDNA pools of two samples followed (30% to 70%) by sequencing on a NextSeq Mid output flowcell.

1.6 Validation of Duplex sequencing

For validation of the liquid biopsy (LB) analysis Seraseq® ctDNA Complete™ Reference Materials (LGC seracare) with 0%, 0.05%, 0.1%, 0.5%, 1% and 5% VAF were used. For validation of the tissue analysis workflow *in silico* dilutions with 8% to 16% VAF of spike-in variants created by spiking 10% and 20% of three internal reference samples into the Ashkenazim Son FFPE Reference Standard NA24385 (SensID) were used. The variants detected above the LOB (LB: 0.25%, tissue: 5%) in processed samples were compared to the trusted regions of the GIAB version 3.3.2 (1) reference data set. The following stretches of low quality in the reference sequence were omitted from the evaluation: chr9:77728672-77728698, chr7:5997427-5997443 and chr8:38428396-38428418. Each variant present in the sample and in the reference data set (true positives, TP), each variant present in the sample only (false positives, FP), and each variant present in the reference data set but not found in the sample (false negatives, FN) were counted to determine sensitivity and positive predictive value (PPV). Sensitivity and PPV were calculated according to the following equations:

Supplemental Equation 1. Determination of sensitivity

$$Sensitivity = \frac{TP}{TP + FN}$$

Supplemental Equation 2. Determination of PPV

$$PPV = \frac{TP}{TP + FP}$$

1.6.1 Determination of trueness

Trueness was determined by calculating the bias for replicate measurements according to the following equation. The mean of reference VAFs in each distinct reference material represents hereby the known VAF of variants.

Supplemental Equation 3. Bias
$\overline{\boldsymbol{x}_{\boldsymbol{meas}}}$: Average of all measurements of a certain VAF; $\boldsymbol{x}_{\boldsymbol{Ref}}$: Reference VAF

$$Bias = \overline{x_{meas}} - x_{Ref}$$

To provide reliable results bias should not exceed 10% leading to trueness >90% (2).

1.6.2 Determination of precision

To assess whether replicate measurements are precise, repeatability of these measurements was assessed by calculating the pooled standard deviation (SD_r).

Supplemental Equation 4. Estimate of random error (repeatability)
SD_r: Estimate of random error; R: number of replicates at each VAF; L: Number of VAFs; r_i: Average result at each VAF; (3)

$$SD_r = \sqrt{\frac{\sum_{i=1}^{L}\sum_{j=1}^{R}[r_{ij} - r_i]^2}{L * (R - 1)}}$$

To provide reliable results precision should be above 80% for replicate measurements of reference materials at the Limit of Quantification (LOQ) (4).

1.6.3 Determination of total error

The total error was calculated according to:

Supplemental Equation 5. Total error
TE: Total Error; SD_S: pooled standard deviation

$$TE = |Bias| + 2 * SD_S$$

According to Little et al. (2) the total error of these measurement results was required to be <50% with a 95% confidence interval at the LOQ (5).

1.7 Karyotyping

Using the Giemsa-trypsin-Giemsa (GTG) banding technique fixed metaphase chromosomes from lymphocyte cultures were stained for karyotyping to assess structural chromosomal abnormalities.

1.8 Microarray

An Infinium® CytoSNP-850K (Illumina) SNP-Array was performed on gDNA from peripheral blood to evaluate copy number variants (CNVs) of ≥18 kb. The SNP-Array was imaged on the iScan Reader (Illumina). Annotated against GRCh38 reference genome and analysis of generated data was performed using the BlueFuse Multi v4.5 software (Illumina). Based on this technology a 562 kb duplication 15q13.3 classified as variant of uncertain significance (VUS) was identified.

1.9 Whole-exome sequencing

Library preparation of gDNA from skin fibroblasts was performed using the Twist Library Preparation Kit (Twist Bioscience, #Cat 104177) as by manufacturers' instructions. 167 ng of gDNA is used as input for mechanical fragmentation on the Covaris E220 system (Covaris) in 8 microTUBE-15 AFA Beads H Slit Strip V2 (Covaris, #Cat 520241) with the following settings: 110 sec treatment with a peak incident power of 18 W, 20% duty factor, and 50 cycles per burst at 20°C. Libraries were quantified using a High Lunatic Plate (Strips D+) (#Cat 7012004) on the Lunatic system (Unchained Labs). Following library preparation target enrichment was performed in accordance with the Twist Target Enrichment protocol (Twist Bioscience) using the Twist Universal Blockers (Twist Bioscience, #Cat 100767), the Twist Hybridization Reagents (Twist Bioscience, #Cat 101026), and the Twist Comprehensive Exome combined with oligos targeting the mitochondrial Genome (Twist Bioscience, #Cat 102033). Prior to hybridization up to eight gDNA samples were pooled equimolarly. Hybridization was performed for 16 h. Pre-pools were quantified using a High Sensitivity DNA Kit (Agilent, #5067-4626) on the Bioanalyzer system (Agilent) and for sequencing five pre-pools were pooled. Paired-end sequencing with 2x151 bp reads was performed on a NovaSeq 6000 system (Illumina). Following sequencing raw data (FASTQ.GZ format) was uploaded to the VARVIS® platform and alignment against the hg19 reference genome and variant calling was performed using the germline bioinformatics pipeline VARFEED worker 1.5.1 with *in silico* validated standard settings.

2 Supplementary Data

2.1 Duplex consensus

Bioinformatics analysis of Duplex sequencing data enables the removal of PCR and sequencing artifacts by identification of the original strand of specific DNA molecules.

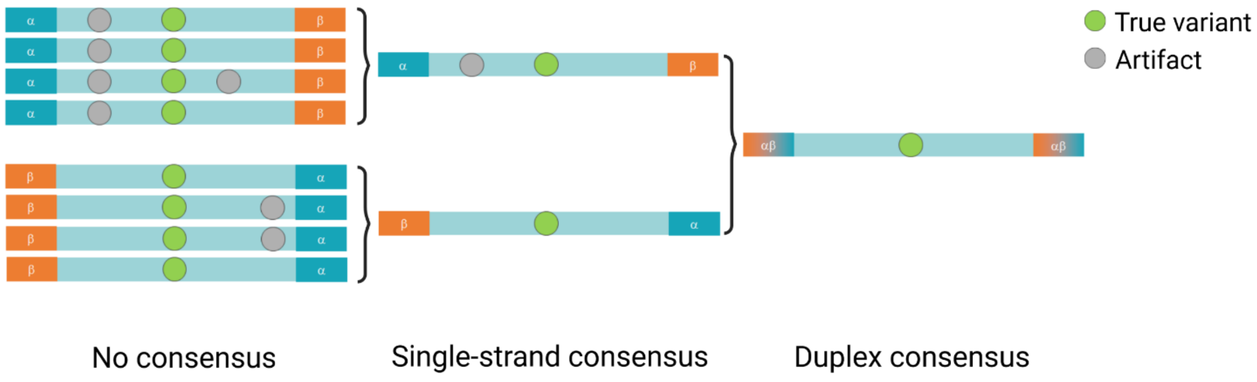

Supplemental Figure 1. Duplex consensus

2.2 Trueness and precision of VAF quantification

To assess the accuracy of VAF quantification with Duplex sequencing for both LB and tissue workflows, measured VAFs were compared to reference VAFs of reference materials (Supplemental Figure 2).

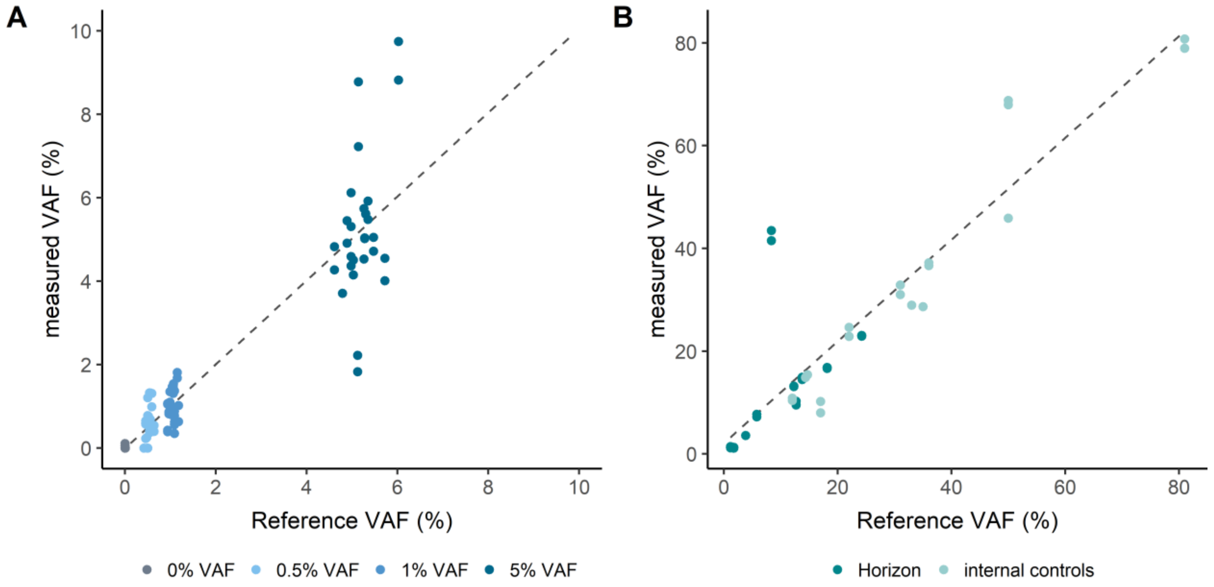

Supplemental Figure 2. Trueness and precision of Duplex sequencing
By comparing the measured VAF of reference samples to the VAF determined by a reference method, both (A) the LB and (B) the tissue analyses were validated.

3 REFERENCES

1. Zook JM, Catoe D, McDaniel J, Vang L, Spies N, Sidow A, et al. Extensive sequencing of seven human genomes to characterize benchmark reference materials. *Sci Data* (2016) **3**:160025. doi:10.1038/sdata.2016.25
2. Little TA. Establishing Acceptance Criteria for Analytical Methods: Knowing how method performance impacts out-of-specification rates may improve quality risk management and product knowledge. *BioPharm International* (2016).
3. CLSI. EP06-A: Evaluation of the Linearity of Quantitative Measurement Procedures: A Statistical Approach; Approved Guideline (2003) **23**.
4. European Medicines Agency. Guideline Bioanalytical method validation.
5. NCCLS. *Protocols for Determination of Limits of Detection and Limits of QuantitationGuideline: Approved Guideline*. NCCLS document EP17-A. NCCLS, 940 West Valley Road, Suite 1400, Wayne, Pennsylvania 19087-1898 USA (2004). 52 p.
